# KSRP Modulation of GAP-43 mRNA Stability Restricts Axonal Outgrowth in Embryonic Hippocampal Neurons

**DOI:** 10.1371/journal.pone.0079255

**Published:** 2013-11-14

**Authors:** Clark W. Bird, Amy S. Gardiner, Federico Bolognani, Daniel C. Tanner, Ching-Yi Chen, Wei-Jye Lin, Soonmoon Yoo, Jeffery L. Twiss, Nora Perrone- Bizzozero

**Affiliations:** 1 Department of Neurosciences, University of New Mexico School of Medicine, Albuquerque, New Mexico, United Sates of America; 2 Department of Biochemistry and Molecular Genetics, University of Alabama at Birmingham, Birmingham, Alabama, United Sates of America; 3 Nemours Biomedical Research, Alfred I. duPont Hospital for Children, Wilmington, Delaware, United Sates of America; 4 Department of Biological Sciences, University of South Carolina, Columbia, South Carolina, United Sates of America; University of Louisville, United States of America

## Abstract

The KH-type splicing regulatory protein (KSRP) promotes the decay of AU-rich element (ARE)-containing mRNAs. Although KSRP is expressed in the nervous system, very little is known about its role in neurons. In this study, we examined whether KSRP regulates the stability of the ARE-containing GAP-43 mRNA. We found that KSRP destabilizes this mRNA by binding to its ARE, a process that requires the presence of its fourth KH domain (KH4). Furthermore, KSRP competed with the stabilizing factor HuD for binding to these sequences. We also examined the functional consequences of KSRP overexpression and knockdown on the differentiation of primary hippocampal neurons in culture. Overexpression of full length KSRP or KSRP without its nuclear localization signal hindered axonal outgrowth in these cultures, while overexpression of a mutant protein without the KH4 domain that has less affinity for binding to GAP-43′s ARE had no effect. In contrast, depletion of KSRP led to a rise in GAP-43 mRNA levels and a dramatic increase in axonal length, both in KSRP shRNA transfected cells and neurons cultured from *Ksrp^+/−^* and *Ksrp ^−/−^*embryos. Finally we found that overexpression of GAP-43 rescued the axonal outgrowth deficits seen with KSRP overexpression, but only when cells were transfected with GAP-43 constructs containing 3′ UTR sequences targeting the transport of this mRNA to axons. Together, our results suggest that KSRP is an important regulator of mRNA stability and axonal length that works in direct opposition to HuD to regulate the levels of GAP-43 and other ARE-containing neuronal mRNAs.

## Introduction

Post-transcriptional mechanisms play a critical role in the dynamic control of gene expression in a number of cellular processes, from cell growth to differentiation. These mechanisms are particularly important in neurons where mRNAs are localized to dendrites and growing axons, and thus can be regulated independently from transcription [1]–[4]. There are multiple stages in the life of an mRNA after transcription, from alternative splicing and stabilization/destabilization to transport and translation. Among these, the control of mRNA stability is one of the least understood processes in neurons (for a review see [5]). The half-life of a transcript is often dependent on the interactions of *cis*-acting mRNA sequences with *trans*-acting factors such as RNA-binding proteins (RBPs) and microRNAs. The best known *cis*-acting destabilizing motif is the AU-rich element (ARE) present in the 3′ untranslated region (UTR) of some short-lived mRNAs such as those for cytokines [6].

In the nervous system, one of the most extensively studied ARE-containing mRNAs is that of the growth associated protein GAP-43. This membrane phosphoprotein is localized to axonal growth cones of developing neurons, where it plays an important role in axon outgrowth and pathfinding [7]–[9]. Precise regulation of GAP-43 expression is essential for correct neuronal function, as demonstrated by the early impairments in axonal targeting and subsequent problems in learning and memory observed in mice lacking GAP-43 [10], [11]. Conversely, overexpression of GAP-43 leads to aberrant axonal sprouting [12]. The RNA-binding protein HuD has been shown in numerous studies to stabilize GAP-43 mRNA both *in vitro* and *in vivo* [13]–[15]. We have previously shown that HuD-mediated stabilization of GAP-43 mRNA leads to increased neurite outgrowth in PC12 cells, primary cortical neuronal cultures and mice overexpressing HuD [15]–[18]. However, until now little has been known about the factors that destabilize the GAP-43 transcript as neurons mature and axonal elongation ceases.

Another RBP expressed in neurons and glial cells is the destabilizing KH-type splicing regulatory protein (KSRP). Originally identified as a DNA Far Upstream Element (FUSE) binding protein 2 (FBP2), KSRP was later shown to enhance splicing of the neuron-specific c-src N1 exon [19], [20]. Other cellular roles of KSRP, also known as chicken ZBP2 and rat MARTA1, were described including transport of MAP-2 and β-actin mRNAs to neuronal processes [21], [22] and promoting microRNA biogenesis [23]. Although multifunctionality is increasingly recognized in RBPs, the best characterized function of KSRP is its ability to destabilize bound ARE-containing mRNAs by targeting them to the exosome for degradation [24], [25]. This has been most extensively studied in the immune system and recently in astrocytes, where KSRP promotes the decay of interleukins, cytokines and inducible nitric oxide (iNOS) mRNAs [24], [26], [27]. Despite the fact that KSRP is expressed at high levels in neurons [28], the function of KSRP in these cells remains poorly understood. In this study we sought to examine the role of KSRP in the control of GAP-43 mRNA levels during neuronal differentiation. We found that KSRP plays an opposite role to HuD, negatively regulating GAP-43 expression and limiting axonal outgrowth.

## Methods

### Plasmids

Control GFP-shRNA and GFP-shKSRP constructs were obtained from SA Biosciences (Qiagen). pAc-GFP-KSRP, pAc-GFP-KSRP 1–4, and pAc-GFP-ΔKH4 plasmids were derived from pET15b-KSRP vector [20]. Coding sequences were amplified using PCR with primers specific to corresponding regions (pAc-GFP-KSRP forward: AAGGCCTCTGTCGACGACTACAGCACGGGAGG, reverse: AGAATTCGCAAGCTTATTCATTGAGCCTGCTGCTGTC; pAc-GFP-KSRP 1–4 forward: AAGGCCTCTGTCGACTCAATGACAGAAGAGTACAGGGTCCCAGA, reverse: AGAATTCGCAAGCTTCTCGATCTTTTCCTCGATAAGCTGCTTGGC; pAc-GFP-ΔKH4 forward: AAGGCCTCTGTCGACTCAATGACAGAAGAGTACAGGGTCCCAGA, reverse: AGAATTCGCAAGCTTCTGGAGGAGGTCGTTGATGATCCGG). pAc-GFP-KSRP plasmid was cloned using Clontech’s In-Fusion enzyme and pAcGFP1-C In-Fusion Ready vector according to manufacturer’s protocols (Clontech). pAc-GFP-KSRP 1–4 and pAc-GFP-ΔKH4 were directionally cloned into the freshly prepared linearized pAc-GFP-KSRP digested with SalI and HindIII. Cloning of GST-KSRP and GST-KSRP-ΔKH4 was described by Gherzi et al [24]. The mCherry-GAP-GAP3’, mCherry-GAP-Amp3’ and mCherry-GAP-γ-actin3’ were cloned into the backbone of the β-actin-mCherry-3′γ-actin using Pfu polymerase to isolate the coding sequence for rat GAP-43 and 3′UTRs of rat GAP-43 and amphoterin mRNAs as previously described [29].

### RNA Electrophoretic Mobility-shift Assay (REMSA)

^32^P-labeled GAP-43 ARE-containing RNA was prepared by *in vitro* transcription from EcoRI linearized pGAP/B plasmid [30], [31] using SP6 RNA polymerase and [α-^32^P] UTP (3000 Ci/mmol, Perkin-Elmer). REMSA assays were performed as previously described [32] with minor modifications. Briefly, 100,000 CPM of ^32^P-UTP labeled RNA was incubated with increasing amounts of purified GST, recombinant GST-KSRP or GST-KSRP-ΔKH4 in a buffer containing 50 mM Tris-HCl pH 7.0, 150 mM NaCl, 0.25 mg/ml tRNA, 0.25 mg/ml bovine serum albumin, and 5% glycerol for 10 min at 37°C. In some experiments, an excess of cold ARE RNA was used to confirm the specificity of the assays. RNA-protein complexes were then run on a non-denaturing 10% polyacrylamide gel in TBE buffer for 45 min. at 200 V. The gel was then dried and exposed to a phosphor screen overnight before radioactivity was measured using a Bio-Rad Personal Molecular Imager FX (Bio-Rad).

### KSRP Cross-Linking Immunoprecipitation (CLIP) Assays

Assays were performed as previously described [33], [34] with minor modifications. Cortices from wild type E17 C57BL/6 mice were dissected and cells triturated before RNA-protein complexes were cross-linked three times with UV light (400 mJ/cm2) using a Stratalinker 2400 (Stratagene). After cross-linking, cells were washed in PBS and lyzed in buffer containing 50 mM Tris-HCl, pH 8.0, 150 mM NaCl, 0.1% SDS, 0.5% deoxycholate, 0.5% TritonX-100, 5 mM NaF, 1 mM Na3VO4, 1 mM EDTA, 1 mM EGTA in the presence of RNase and protease inhibitors. Lysates were pre-cleared by centrifugation and incubated with Protein G Dynabeads (Life Technologies) pre-bound with anti-KSRP/FBP2 (Novus Biologicals,), or non-immune IgG for 2 hours at 4°C with rotation. Immunoprecipitates were washed once with lysis buffer, once with low salt buffer (20 mM Tris-HCl, pH 8.0, 150 mM NaCl, 0.1% SDS, 0.5% TritonX-100, 2 mM EDTA), and once with high salt buffer (20 mM Tris-HCl, pH 8.0, 500 mM NaCl, 0.1% SDS, 0.5% TritonX-100, 2 mM EDTA) for 10 minutes at 4°C with rotation. Samples were then treated with DNase I (Promega) for 30 minutes at 37°C followed by Proteinase K treatment. RNA was extracted with Trizol (Life Technologies) and used for qRT-PCR.

### Competitive RNA Binding Assay

Protein G Dynabeads were washed and pre-bound with HuD E-1 antibody (Santa Cruz). ^32^P-labeled GAP-43 ARE containing RNA was prepared as described above. Labeled GAP-43 ARE was incubated with 1.5 nmol GST-HuD protein and increasing amounts of GST or GST-KSRP in a binding buffer containing 10 mM HEPES pH 7.4, 100 mM KCl, 5 mM MgCl_2_, 0.5% NP40 and along with 40 U of RNasin™ RNase inhibitor (Promega). Assays were then incubated for 10 minutes at 4°C before being exposed to UV light for 30 minutes at 4°C. Pre-bound beads were then added to the binding assays and then mixed at 4°C for 1 hour. After washing three times with binding buffer, samples were resuspended in Tris-EDTA buffer and the radioactivity measured by scintillation counting.

### *In vitro* mRNA Decay Assay

S100 extracts were prepared from cortical brain tissue of adult *Ksrp* ^−/−^ mouse [35] as previously described [36]. GST protein and recombinant GST-KSRP or GST-KSRP-ΔKH4proteins was expressed in BL21 *E. coli* and purified using the MagneGST™ Protein Purification System (Promega) according to the manufacturer’s protocol. ^32^P-labeled GAP-43 ARE containing RNA (pGAP/B, [31]) and a non-ARE containing control RNA (pGAP/C, [31]) were capped and polyadenylated as previously described [13]. The non-ARE containing RNA derived from the C region of GAP-43 3′ UTR (nt 795–915) was selected as control RNA since we have previously shown that mRNAs containing this 3′ UTR region are very stable [31]. Decay reactions were performed as described by Bolognani et al [15], with minor modifications: 50 fmol ^32^P-labeled GAP-43 RNA was incubated with 20 µg S100 protein and 50 ng purified GST or recombinant GST-KSRP and GST-KSRP-ΔKH4 proteins.

### Real-time RT-PCR

Quantitative real-time RT-PCR (qRT-PCR) was performed as described previously [18]. Briefly, cDNA was synthesized using 1 µg total RNA using Superscript II reverse transcriptase (Invitrogen) according to manufacturer’s protocol. Exon spanning primer pairs were designed using Primer Express 3.0 (Life Technologies) and validated using NCBI primer BLAST software. Primers used for analysis were: *KSRP* (forward 5′-GGACTCAGGCTGCAAAGTTC, reverse 5′-CCAGGATCATCTTTGCCTTT), *GAP-43* (forward 5′-AGCCAAGGAGGAGCCTAAAC, reverse 5′-CTGTCGGGCACTTTCCTTAG), and *GAPDH* (forward 5′-TGTGATGGGTGTGAACCACGAGAA, reverse 5′-GAGCCCTTCCACAATGCCAAAGTT). qRT-PCR reactions were performed on an Applied Biosystems 7300 Real Time PCR System. Gene expression levels were analyzed using SYBR Green (Life Technologies). No evidence of primer dimerization was evident by dissociation curve analysis. Primers for KSRP and GAP-43 were validated against GAPDH and were within optimal amplification values (slope <|0.1|). Samples were run in triplicate, and relative levels of expression compared to GAPDH were calculated using the 2^−ΔΔCt^ method [37]. The specificity of the assays for RNA was validated using a PCR reaction without reverse transcriptase (No RT control).

### Cell Culture

Primary hippocampal neuronal cultures were isolated and grown as described previously [38]. Briefly, hippocampi from E17 Sprague-Dawley rats or E17 *Ksrp* +/+, *Ksrp* +/− and *Ksrp* −/− mouse embryos [35] were pooled, triturated with a fire-polished Pasteur pipette and dissociated neurons plated on poly-D-lysine/laminin coated coverslips (BD Biosciences, San Jose, CA). Cultures were grown in Neurobasal medium (Life Technologies) supplemented with B27 supplement (Life Technologies), Penicillin/Streptomycin, 0.5 mM L-glutamine and 25 µM glutamate for 3 days *in vitro* (DIV) before being transfected with GFP plasmid constructs using Lipofectamine 2000™ and Opti-MEM (Life Technologies) according to manufacturer’s protocol. Cells were incubated with transfection medium for 48 hours before being fixed with 4% paraformaldehyde. All procedures using animals were approved by the University of New Mexico Institutional Animal Care and Use Committee (IACUC) in compliance with the Guidelines for the Care and Use of Laboratory Animals established by the National Institutes of Health (NIH).

PC12 cells were maintained in RPMI-1640 media supplemented with 7.5% horse serum and 2.5% fetal calf serum at 37°C, 5% CO2 as previously described [16]. These cells were transfected with KSRP shRNA and control non-targeting constructs using Lipofectamine 2000™ as described above.

### Immunocytochemistry

Fixed primary hippocampal cultures were incubated with 50 mM ammonium chloride in phosphate-buffered saline (PBS) for 20 minutes to quench paraformaldehyde autofluorescence. The cells were then incubated with 1% horse serum and 0.1% Triton X-100 in PBS (PBST) for 30 minutes. Neurofilament light chain (NF-L) was detected with α-NF-L antibody generated in mouse (MAB1615, Millipore) at a 1∶100 concentration. KSRP was detected using α-FBP-2 antibody generated in goat (SC-33031, Santa Cruz Biotechnology) at a 1∶100 concentration. Cells were incubated with primary antibody for 2 hours at room temperature before being rinsed with PBST. Alexa-Fluor 546 donkey α-mouse antibody or Alexa-Fluor 546 donkey α-goat antibody (Life Technologies) was then incubated with the cells in PBS containing 1% horse serum for 1 hour at room temperature in the dark at a concentration of 1∶200. The cells were then rinsed with PBST before coverslips were mounted onto glass slides (VWR) with mounting media containing polyvinyl alcohol with DABCO anti-fading reagent (PVA-DABCO, Sigma).

### Microscopy and Image Analysis

For quantitation of axonal outgrowth, transfected neurons were imaged with an Olympus BX60 fluorescence microscope with a 20X objective and images collected using an Olympus DP71 camera. For neurons that were too large to be imaged in a single 20X field, multiple overlapping images of the same neuron were taken and the images merged together using Adobe Photoshop Elements (Adobe Systems Inc.). For quantitation of KSRP shRNA knockdown or KSRP overexpression, transfected primary hippocampal neurons were imaged with a Zeiss LSM 510 confocal microscope. Axonal outgrowth of transfected primary hippocampal neurons was measured using Neurolucida (MBF biosciences). Cell bodies and primary axons were traced using the program, and overall axonal length per neuron was calculated. Slides were codified and analyses were performed blind with regards to the treatment. Axonal length was determined using multiple transfection experiments run in duplicates. In each coverslip, roughly 6–8 GFP positive neuron axons were measured for overall length, and these values were averaged together to generate a single n per slide for statistical purposes. About 50 cells were measured per condition over at least 6 separate culture preparations for each construct.

### Quantitation of KSRP Protein Knockdown by shRNA

E17 rat hippocampal cultures were grown as described above, and then transfected with GFP-shKSRP or control GFP-shRNA plasmids at 3 DIV. These cells were allowed to grow for another 48 hours *in vitro* before being fixed and immunostained with α-KSRP antibodies. Transfected cells were analyzed for KSRP protein expression by confocal microscopy. KSRP expression levels were quantitated by comparing KSRP immunofluorescence in a transfected neuron vs. untransfected cells in the same image. Fluorescence intensity was measured using ImageJ (NIH).

### Fluorescence Activated Cell Sorting

Fluorescence activated cell sorting (FACS) was performed in the UNM Flow Cytometry Facility by dedicated personnel. PC12 cells were incubated overnight in Opti-MEM media supplemented with 4% serum before being transfected with either control non-targeting or shKSRP GFP plasmids (Origene). Cells were grown for 48 hours before they were trypsinized and pelleted, and then resuspended in sorting medium (cation free PBS supplemented with 0.2% fetal bovine serum, 10 mM HEPES pH 7.3, 1 mM EDTA) at a concentration of 5×10^6^ cells/ml. Cells were then sorted, using gating to collect the brightest 3% of GFP positive cells. Cells were collected in sorting medium, pelleted by centrifugation and flash frozen on dry ice before RNA was extracted using RNAeasy (Qiagen) and analyzed by qRT-PCR.

### Statistics

ANOVA with Tukey’s multiple comparison test and Student’s t-tests were performed using GraphPad Prism v. 6.0 software (GraphPad Software, Inc.).

## Results

### KSRP Binds to GAP-43 mRNA *in vitro*

**Figure 1:**
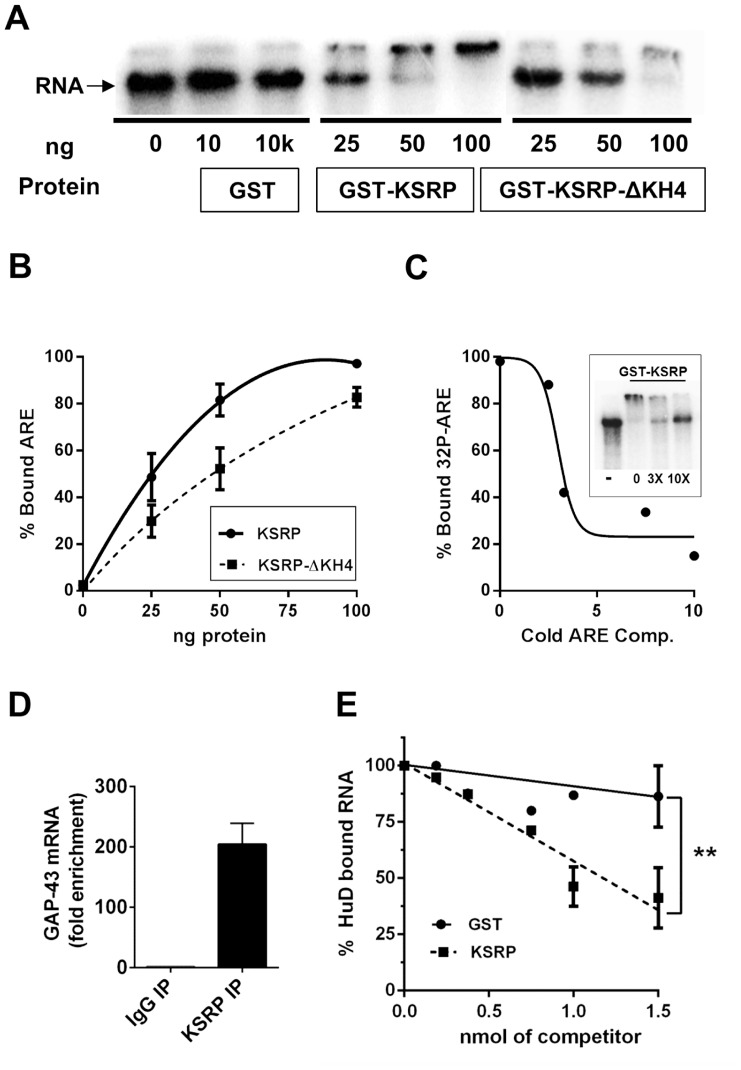
Binding of KSRP to GAP-43 ARE. **A**. REMSA assays with increasing amounts of purified GST, GST-KSRP, or GST-KSRP-ΔKH4 protein and ^32^P-labeled GAP-43 ARE. Arrow shows the migration of unbound RNA **B**. Binding curves comparing the relative affinities of the GST-KSRP and GST-KSRP-ΔKH4 for the GAP-43 ARE. Results are the average of 3 separate experiments. **C**. Displacement of bound radiolabeled GAP-43 ARE from GST-KSRP in the presence of excess of cold competitor. **D**. Enrichment of GAP-43 mRNA precipitated using KSRP antibodies vs. control IgG following *in vivo* UV-crosslinking immunoprecitation (CLIP) assays. **E**. Competitive binding assay of HuD and KSRP. ^32^P-labeled GAP-43 ARE was incubated with HuD and increasing amounts of either GST or KSRP competitor protein, before HuD was pulled down and bound RNA measured by scintillation counting. **p<0.01 for difference between the two lines (GST, y = −10x+100 vs. KSRP, y = −44x+100) by Fisher’s r-to-z-test.

Given that KSRP is known to bind ARE sequences, initial studies used two different RNA-protein binding assays to determine if this RBP binds to GAP-43 mRNA. First, we utilized an RNA electrophoretic mobility shift assay (REMSA) using radiolabeled GAP-43 ARE. As shown in Figure 1A, incubation with 25 ng of GST-KSRP protein was sufficient to cause a shift in the migration of a GAP-43 ARE containing RNA, and bands were completely shifted in the presence of 100 ng KSRP protein. In contrast, there was no shift in the mobility of the mRNA when incubated with GST, even when incubated with up to 10 µg of GST protein. Previous research showed that KSRP binds to ARE sequences primarily via its fourth KH domain [24]. Consistent with this finding, KSRP protein lacking the KH4 domain (GST-KSRP-ΔKH4) displayed reduced binding to the GAP-43 ARE, and higher levels of recombinant protein were required to visualize a mobility shift than did wild type KSRP (Figure 1A and B). The specificity of the interaction of KSRP with GAP-43 ARE was confirmed by displacement of the radiolabeled RNA with an excess of cold ARE competitor (Figure 1C). Finally, binding of endogenous KSRP to GAP-43 mRNA *in vivo* was confirmed using UV-cross-linking immunoprecipitation (CLIP) assays [33], which showed a near 200-fold enrichment of this mRNA in the KSRP IP relative to control IgG (Figure 1D).

Given that both KSRP and HuD [13] bind to the same ARE sequence in the GAP-43 3′ UTR, we then examined the ability of KSRP to compete with HuD for the binding of this RNA. For these studies, ^32^P-labeled GAP-43 ARE was incubated with 1.5 nmol of GST-HuD protein and increasing amounts of either GST or GST-KSRP. HuD was then immunoprecipitated and the amount of RNA bound was measured by scintillation counting. Increasing amounts of GST protein did not interfere with HuD binding to GAP-43 ARE, while increasing amounts of KSRP significantly displaced HuD from the ARE-containing RNA (Figure 1E). Furthermore, when equal molar amounts of KSRP and HuD were used in the assay HuD binding to GAP-43 ARE decreased by roughly half, suggesting that KSRP and HuD have similar affinities for binding the GAP-43 ARE.

### KSRP Enhances GAP-43 mRNA Decay

**Figure 2:**
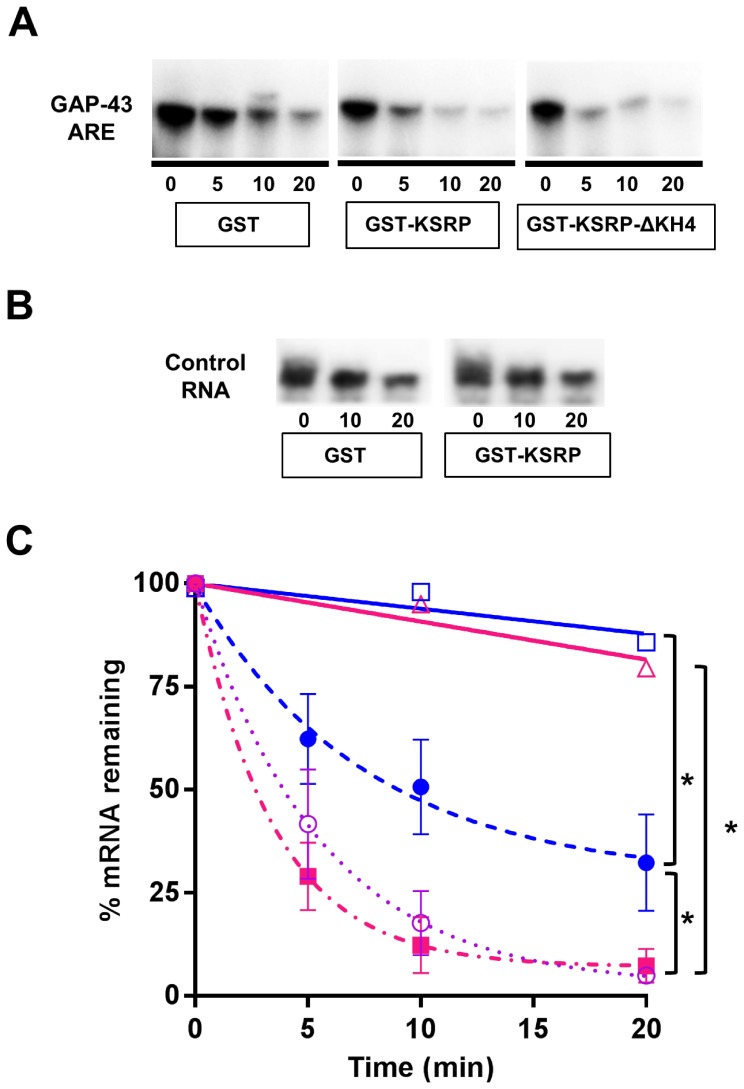
KSRP increases GAP-43 ARE decay *in vitro.* **A**. Representative images from mRNA decay assays. Purified recombinant GST, GST-KSRP, or GST-KSRP-ΔKH4 protein was incubated with ^32^P-labeled GAP-43 ARE in the presence of S100 extracts from *Ksrp^−/−^* mice. **B**. In vitro decay of a control GAP-43 RNA that does not contain the ARE and is stable even in the presence of KSRP. **C**. Decay curves showing the results of 3 separate decay experiments fitted with a single rate exponential decay curve. Comparisons of the different decay rates were performed by Two-Way ANOVAs after transforming the data to *ln*: *p<0.05 ARE/GST vs. ARE/GST-KSRP[F (1, 20) = 4.65333, p = 0.04334]; ARE/GST vs.Co/GST [F (1,14) = 6.2256, p = 0.02571] and ARE/GST-KSRP vs. Co/GST-KSRP [F (1,14) = 6.88683, p = 0.02001].

To determine if KSRP has an effect on GAP-43 mRNA stability, subsequent experiments used an *in vitro* RNA decay assay that we have previously utilized to demonstrate the role of HuD in the stabilization of this mRNA [15], [39] (Figure 2). This experimental system has been shown to reliably reproduce the relative decay rates of several mRNAs, as all the components of protein complexes required for mRNA degradation are present in S100 extracts [36], [40]. In these assays, ^32^P-labeled capped and polyadenylated RNA containing the GAP-43 ARE was incubated with purified recombinant GST-KSRP or GST- KSRP-ΔKH4 proteins along with S100 protein extracts from *Ksrp^−/−^* mice [35] and decay of the labeled mRNA was measured over time. The use of S100 extracts from *Ksrp^−/−^* mice also ensures the absence of endogenous KSRP, which could confound the decay results. When GST was incubated with GAP-43 ARE, the mRNA decayed with a half-life of about 10 minutes. Addition of GST-KSRP to the decay system significantly enhanced the degradation rate of GAP-43 ARE, decreasing the half-life of the mRNA to 3 minutes as seen for other ARE-containing mRNAs [24]. In contrast when the truncated version of KSRP (GST-KSRP-ΔKH4) was used in the decay assay the half-life of the mRNA was about 7 min, an intermediate value between the other two conditions. To control for the specificity of the decay assays for the GAP-43 ARE, additional decay assays were performed using an RNA derived from non-ARE region of the GAP-43 3′UTR (Figure 2B and C), which was found to be very stable even in the presence of KSRP (Figure 2B and C). Also, we confirmed that the yield of RNA recovered from the assays was the same for all the time points, using a “spike-in” radiolabeled control RNA in addition to the GAP-43 mRNA (Figure S1). Collectively, these experiments indicate that KSRP increases the decay rate of GAP-43 mRNA *in vitro* and that the fourth KH domain in the protein is important for this function.

### KSRP Limits Axonal Outgrowth in Cultured Rat Hippocampal Neurons

**Figure 3:**
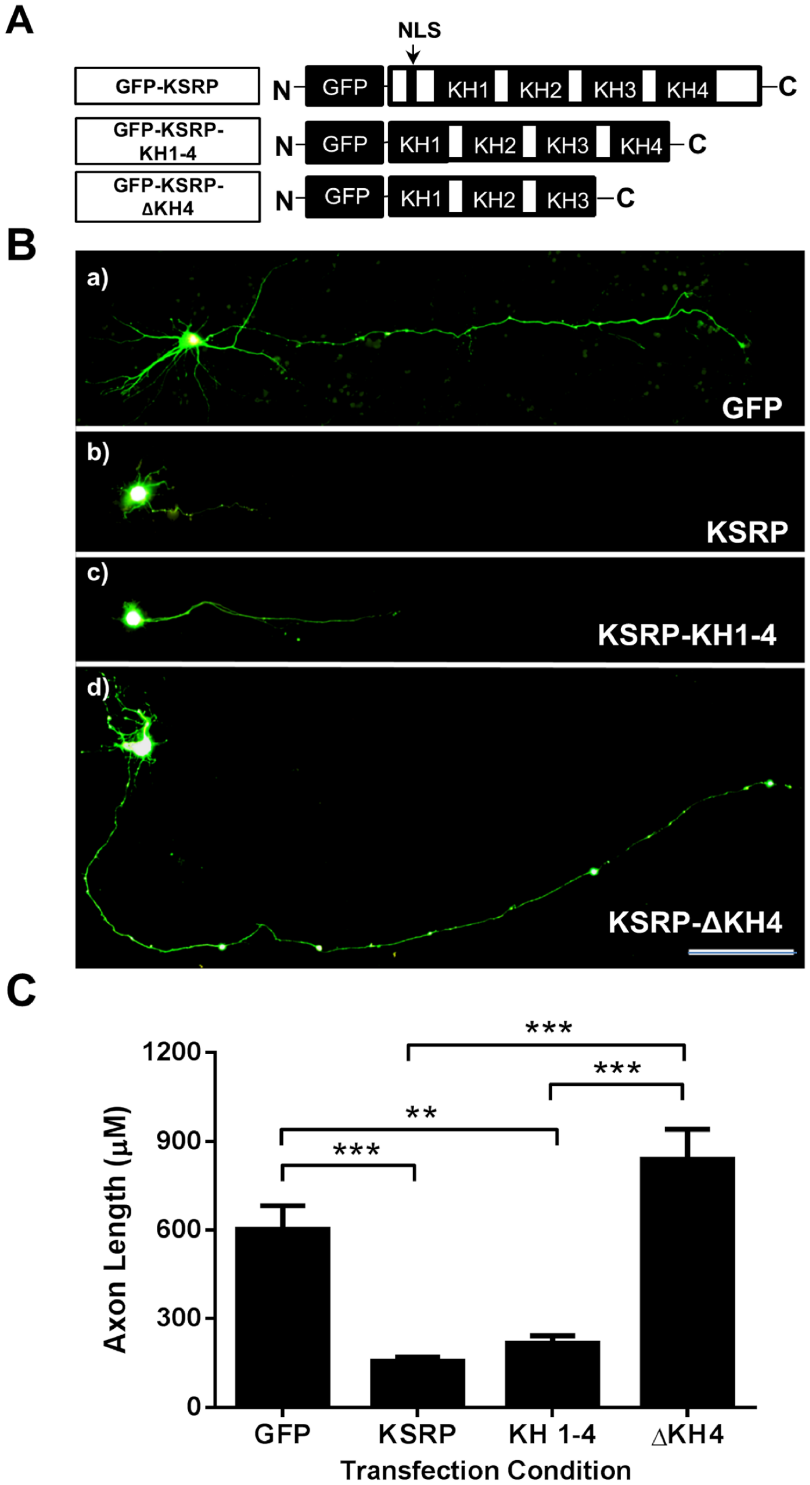
Overexpression of KSRP limits axonal outgrowth in hippocampal neurons. **A**. GFP-KSRP constructs cloned for KSRP overexpression studies. GFP-KSRP-KH 1–4 lacks a NLS, while the GFP-KSRP-ΔKH4 lacks both a NLS and the fourth KH domain. **B**. Rat hippocampal neurons transfected with KSRP constructs. Transfection conditions: a) GFP, b) KSRP, c) KSRP KH 1–4, d) KSRP ΔKH4. Scale bar is 100 µm. **C**. Quantitation of axonal outgrowth in KSRP transfected hippocampal neurons. Averaged axonal outgrowth from several transfection experiments is shown (mean +/− SEM). **, p<0.01; ***p<0.001 using a One way ANOVA with Tukey’s multiple comparison post-hoc tests (GFP n = 8, KSRP n = 11, KSRP KH 1–4 n = 6, KSRP ΔKH4 n = 10).

Having established that KSRP binds to and affects GAP-43 mRNA half-life, we set out to determine the potential physiological effect(s) of KSRP expression in cultured rat hippocampal neurons. In this experimental system hippocampi from E17 rat embryos were isolated, dissociated, and grown for 3 DIV before being transfected with various GFP-KSRP constructs (Figure 3A). Neurons were grown for 48 hours post-transfection before fixation. After fixing, cells were immunostained with NF-L antibody to confirm that the transfected cells being observed were actually neurons and not astrocytes (data not shown).

The effect of overexpression of isoforms of KSRP is shown in Figure 3B–C. Neurons transfected with control GFP vector had an average axonal length of c. 600 µm (Figure 3C). When neurons were transfected with GFP-KSRP axonal outgrowth was significantly stunted, with neurons having an average axonal length of c. 150 µm (Figure 3B–C), which is significantly different from GFP transfected cells. KSRP was originally identified to function in enhancing mRNA splicing, so to eliminate the possibility that changes in axonal outgrowth were due to splicing effects rather than stability effects, we transfected neurons with a KSRP construct (GFP-KSRP-KH 1–4) encoding a protein that lacks a nuclear localization signal and it does not concentrate in the nucleus [41]. This construct also significantly limited axonal outgrowth in our cultured hippocampal neurons (average length of c. 200 µM), suggesting that KSRP’s role in mRNA splicing does not contribute to its effects in axonal outgrowth. However, overexpression of GFP-KSRP-ΔKH4, which has reduced affinity for GAP-43 ARE (Figure 1A and 1B), did not limit axonal outgrowth when compared to control GFP transfected cells and resulted in significantly longer axons than GFP-KSRP and GFP-KSRP-KH 1–4 transfected neurons (Figure 3C). Together, these data suggest that KSRP limits axon growth through binding to cytoplasmic RNAs via its fourth KH domain.

### Knockdown of KSRP Enhances Axonal Outgrowth in Hippocampal Neurons

**Figure 4:**
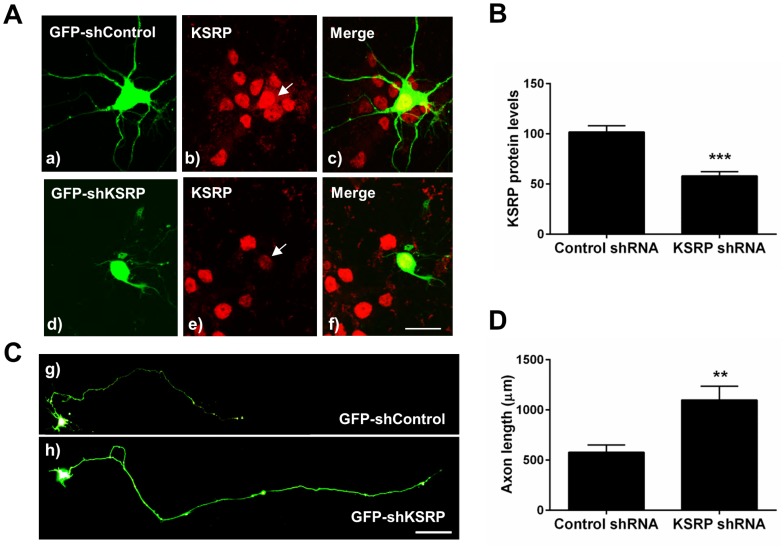
shRNA knockdown of KSRP increases axonal length in transfected hippocampal neurons. **A**. Representative images of shRNA transfections. Control (a–c) or KSRP (d–f) shRNA plasmids were transfected into primary hippocampal neuronal cultures, and KSRP expression was quantified by comparing KSRP immunofluorescence levels with untransfected cells in the same field. Arrows point to the nuclei of transfected cells. Scale bar is 25 µm. **B**. Quantification of KSRP knockdown by GFP-KSRP-shRNA. Knockdown was measured by comparing KSRP immunofluorescence intensity in the nuclei of shRNA transfected cells vs. untransfected cells in the same image frame. Plotted graphs are relative mean (+/− SEM) levels of KSRP fluorescence intensity. **, p<0.01, Student’s t-test (n = 7 for control shRNA; n = 14 for KSRP shRNA). **C**. Rat hippocampal neurons transfected with shRNA constructs. Transfection conditions: g) Non-targeting GFP-shRNA control vector or h) GFP-KSRP-shRNA. Scale bar is 100 µm. **D**. Quantitation of axonal outgrowth in shRNA transfected hippocampal neurons. Averaged axonal outgrowth from 10 separate transfection experiments is shown (mean +/− SEM). **p<0.01 using Student’s t-test.

To further characterize the effect of KSRP expression on axonal outgrowth, we depleted KSRP from cultured hippocampal neurons using a GFP expressing shRNA construct. Decrease in KSRP protein was confirmed by comparing immunofluorescence intensity of KSRP in the nucleus of shRNA transfected cells versus untransfected cells in the same field. Expression of shKSRP reduced KSRP protein levels by approximately 50% (Figure 4 A and C). In contrast, control (non-targeting) shRNA transfected cells had the same KSRP immunofluorescence intensity when compared to other cells in the same image frame.

**Figure 5:**
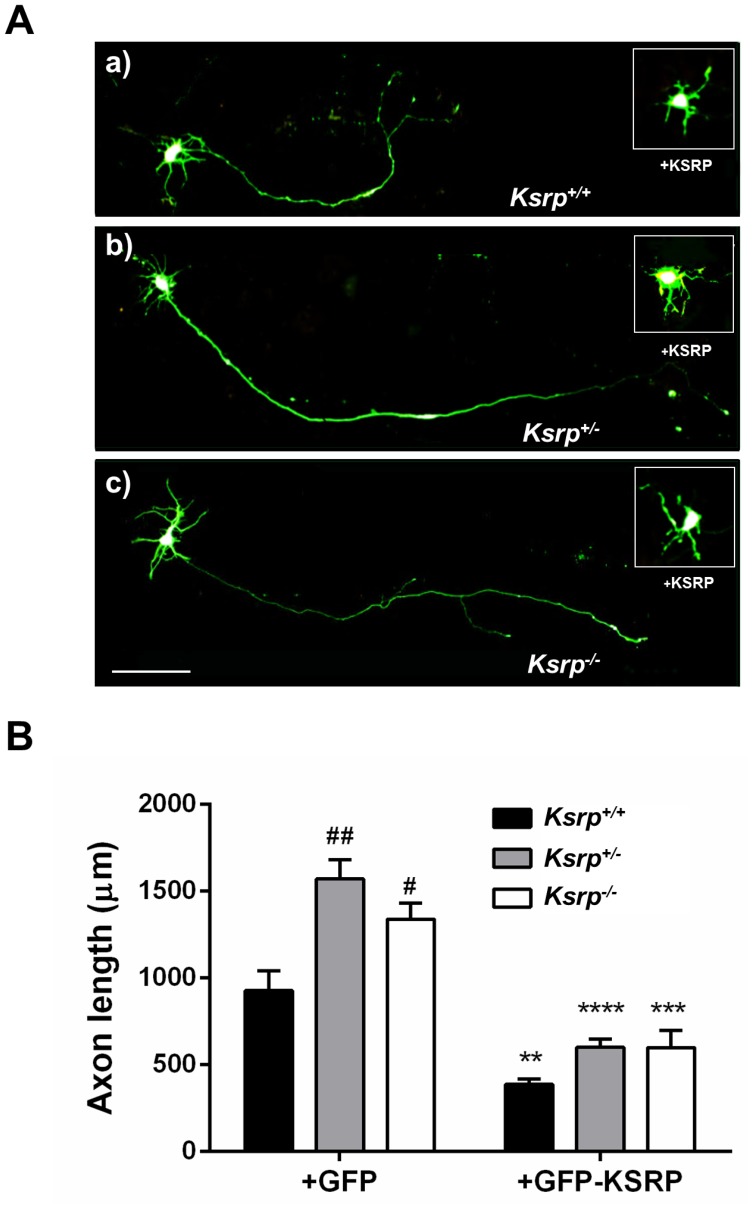
Increased axonal outgrowth in *Ksrp^+/−^* and *Ksrp^−/−^* neurons is hampered by KSRP overexpression. **A**. Representative images of E17 cultured hippocampal neurons from wild type (WT), KSRP heterozygous and KO embryos transfected with either control GFP (a–c) or GFP-KSRP (insets in a–c) plasmids. Scale bar is 100 µm for both main panels and insets. **B**. Average axonal length (mean +/− SEM) from 2–3 separate cultures of 7–9 embryos in each genotype transfected with either GFP or GFP-KSRP (n = 10–15 cells per culture). Two way ANOVA results: interaction [F (2, 26) = 2.961, p = 0.0694], genotype [F (1, 26) = 111.9, p<0.0001], and transfection condition, [F (2, 26) = 12.69, p = 0.0001]. One way ANOVA and Tukey’s multiple comparison tests demonstrate effect of genotype ## p<0.01 *Ksrp^+/−^* vs. *Ksrp^+/+^,* and # p<0.05 *Ksrp^−/−^* vs. *Ksrp^+/+^,* and effect of GFP-KSRP transfection **p<0.01, ****p<0.0001 and ***p<.0.001 for *Ksrp^+/+^, Ksrp^+/−^* and *Ksrp^−/−^*, respectively.

The effect of KSRP on axonal elongation was measured in the same manner as the KSRP overexpression studies, using the same time course, and NF-L counterstaining to confirm that neurons were being examined. When hippocampal neurons were transfected with non-targeting control GFP-shRNA (Figure 4 B and D), axons grew to an average length of c. 580 µm, which is not significantly different from GFP transfected cells (Figure 3C). Depletion of KSRP resulted in axons approximately twice as long as control shRNA transfected neurons (average of c. 1100 µm, Figure 4D). Similar increases in axonal length were also seen with second shKSRP construct targeting a different sequence in KSRP (Figure S2). Furthermore, using cultured neurons from *Ksrp^+/−^* and *Ksrp^−/−^* mouse embryos we found that depletion and complete loss of KSRP in these neurons led to significant increases in axonal growth (Figure 5). Western blots validated the absence of KSRP protein in the *Ksrp* ^−/−^ cortical tissues and significant knockdown in the *Ksrp* ^+/−^ to about 15% of the levels in wild type mice (Figure S3). Since the levels of KSRP in the heterozygous mice are very low, we did not observe any significant differences in axonal outgrowth between them and the complete knockout animals (Figure 5B). Additionally, we found that the increased axonal growth seen in *Ksrp* ^+/−^ and *Ksrp^−/−^* neurons was completely reversed by transfection with a KSRP expression plasmid (Figure 5A; a-c insets and Figure 5B). Altogether, these data indicate that even partial depletion of KSRP results in enhanced axonal length, further implicating KSRP as an important regulator of axogenesis.

### Increased GAP-43 mRNA Levels after *in vivo* Knockdown of KSRP

**Figure 6:**
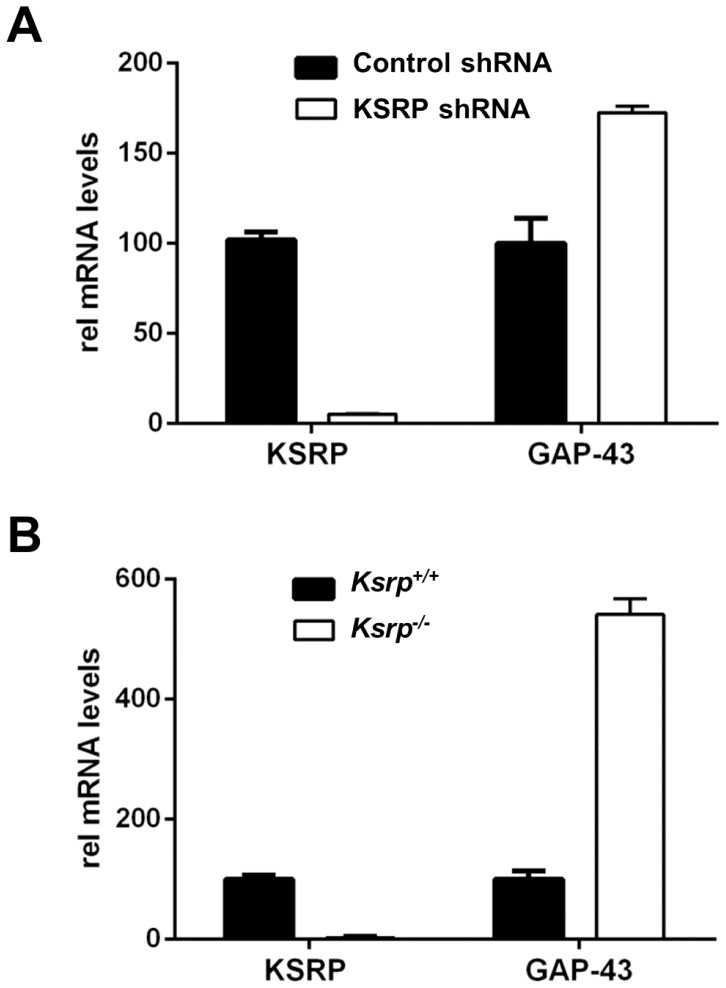
Increased levels of GAP-43 mRNA in PC12 cells with reduced KSRP expression and in E17 cortices from *Ksrp^−/−^* mice. **A**. KSRP mRNA knockdown and GAP-43 upregulation in PC12 cells transfected with pGFP-shKSRP relative to control non-targeting pGFP-shRNA. GFP expressing transfected PC12 cells were enriched by FACS to collect the brightest 3% fraction of cells before using them for KSRP and GAP-43 qRT-PCR. **B**. RNA was extracted from E17 *Ksrp^+/−^* and *Ksrp^−/−^* cortical tissue. *Ksrp^−/−^* cortices contained significantly greater levels of GAP-43 mRNA.

Given that GAP-43 is an important regulator of axonal growth, we tested the effect of KSRP knockdown on GAP-43 mRNA levels *in vivo* using PC12 cells and E17 *Ksrp* ^−/−^ cortices. PC12 cells were transfected with GFP-shKSRP and GFP-control non-targeting shRNA, and incubated for 48 hours before they were purified using FACS to enrich for the brightest 3% of transfected GFP positive cells. Analysis of KSRP and GAP-43 mRNA expression by qRT-PCR (Figure 6A) revealed that knockdown of KSRP by the shRNA construct was robust; lowering KSRP mRNA levels by roughly 95%. Under these conditions, GAP-43 mRNA levels were increased 1.7-fold (Figure 6A). Similarly, GAP-43 mRNA levels were found to be increased in cortices from E17 *Ksrp* ^−/−^ mice by more than 5-fold (Figure 6B). These results indicate that KSRP is a negative regulator of GAP-43 expression, as knockdown of KSRP can increase GAP-43 expression both in culture and *in vivo*.

### Rescue of Limited Axonal Outgrowth in KSRP Transfected Neurons by Co-expression of GAP-43

Based on the effects of overexpression versus knockdown of KSRP upon axonal outgrowth, and the knowledge that KSRP binds to and affects the stability of GAP-43 mRNA, we sought to determine if the phenotype resulting from KSRP overexpression could be rescued by overexpressing GAP-43 in the same neurons. For this, we co-transfected hippocampal neurons with GFP-KSRP and varying GAP-43 expression constructs. Since GAP-43 mRNA is known to localize into axonal processes through ARE in its 3′UTR [42], we used different 3′UTR sequences to restrict GAP-43 mRNA to the cell body or target it into axons. The mCherry-GAP-GAP3’ plasmid contains GAP-43 coding region along with GAP-43 3′UTR, which contains the ARE and the element necessary for targeting GAP-43 to axons [42], where its localized translation is required for promoting axonal extension. mCherry-GAP-AMP3’ contains the GAP-43 coding region with the 3′UTR from amphoterin, which does not contain an ARE but does contain an axonal targeting sequence and similarly increases axonal growth [29]. As control we used mCherry-GAP-γ-actin3’, which has the GAP-43 coding region with γ-actin 3′UTR, and does not contain an ARE or an axonal targeting sequence.

**Figure 7:**
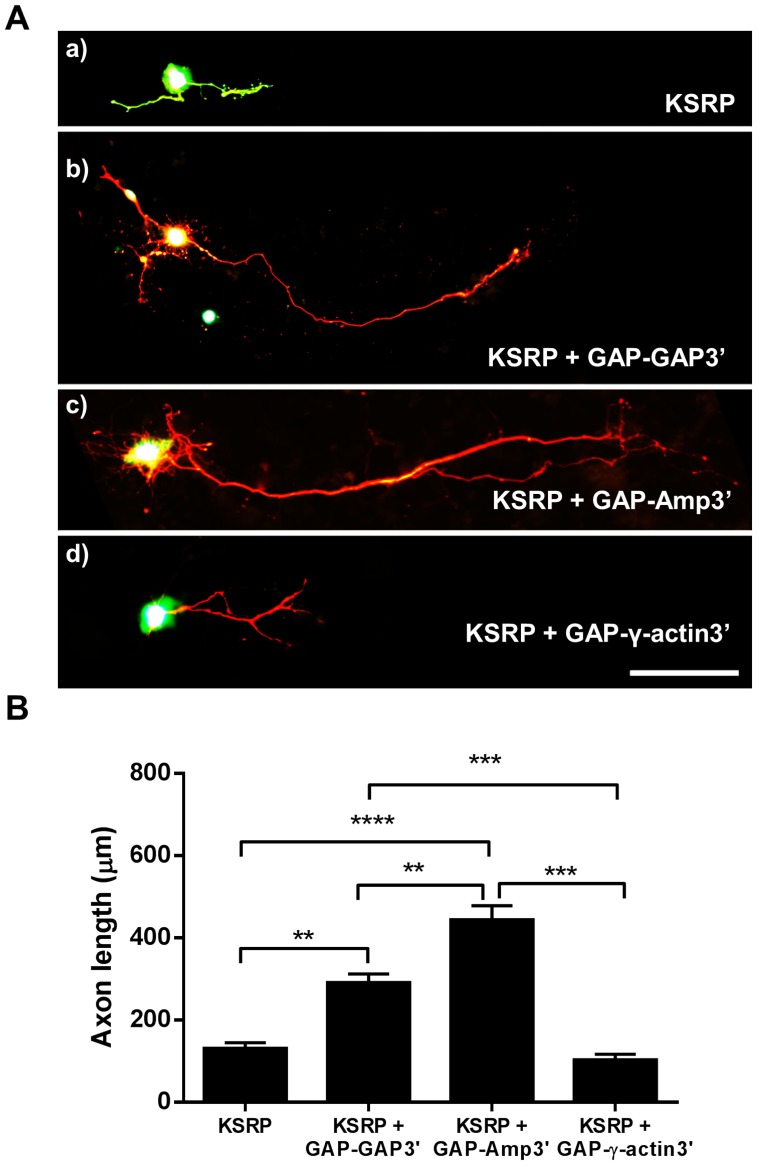
Overexpression of GAP-43 rescues limited axonal outgrowth in KSRP transfected hippocampal neurons. **A**. Rat hippocampal neurons transfected with GFP-KSRP and various mCherry-GAP-43 (mCherry-GAP) constructs with different 3′ UTRs. Transfection conditions: a) GFP-KSRP only, b) GFP-KSRP+mCherry-GAP-GAP3’, c) GFP-KSRP+mCherry-GAP-Amp3’, d) GFP-KSRP+mCherry-GAP-γ-actin3. Scale bar is 100 µM. **B**. Quantitation of axonal outgrowth in transfected neurons. Averaged data from at least 5 neurons per transfection from 6 separate transfection experiments is shown (mean +/− SEM). **p<0.01, ***p<0.001 and ****p<0.0001 using One way ANOVA with Tukey’s multiple comparison post-hoc tests.

Hippocampal neurons transfected with both mCherry-GAP-GAP3’ and GFP-KSRP had axons grow to lengths significantly longer than neurons transfected with GFP-KSRP alone (c. 300 µm vs. 150 µm, Figure 7). Although mCherry-GAP-GAP3’ mRNA is susceptible to KSRP-mediated degradation, the strong CMV promoter driving its expression is likely able to overcome the 3-fold increase in GAP-43 mRNA turnover mediated by KSRP, leading to increased axonal outgrowth relative to KSRP alone. Consistent with the lack of ARE in the Amphoterin 3′ UTR, rescue experiments using mCherry-GAP-AMP3’ were more efficient than with mCherry-GAP-GAP3’ as neurons grew significantly longer axons (c. 450 µm) than those transfected with mCherry-GAP-GAP3’ and much longer than those transfected with GFP-KSRP (Figure 7B). In contrast, mCherry-GAP-γ-actin3’ did not rescue axonal outgrowth in KSRP transfected cells, indicating that targeting of GAP-43 mRNA to axons is required for its effect on axonal elongation.

## Discussion

RNA-binding protein-mediated mRNA stabilization is an important mechanism for controlling gene expression during cell growth and differentiation. This is especially true in the nervous system, where the neuronal RBP HuD was shown to stabilize the mRNAs of several growth related genes, such as GAP-43, contributing to neuron development and function [5]. Despite the evident role of these post-transcriptional mechanisms in neurons, very little is known of the factors responsible for destabilizing neuronal mRNAs. This could be particularly relevant during development and cellular maturation to remove mRNAs as the neuron reaches a stage where growth-associated proteins like GAP-43 are no longer needed. In this study, we have identified KSRP as a negative regulator of GAP-43 mRNA stability in developing neurons. Our data indicate that KSRP functions in an opposite manner to HuD by promoting mRNA decay and limiting axonal outgrowth.

*In vitro* studies examining how KSRP interacts with GAP-43 mRNA clearly indicate that KSRP binds to the highly conserved type III ARE present in GAP-43 3′UTR, which is required to destabilize the transcript [13]. Our data show that KSRP and HuD further compete for binding to the same site in GAP-43 mRNA with similar affinity. However, binding of KSRP to the GAP-43 ARE has the opposite effect of HuD, decreasing mRNA stability by almost 3 fold. While *in vitro* work is important in demonstrating KSRP modulation of GAP-43 transcript stability, there are a number of other factors that interact in cells to affect gene expression. Supporting this *in vitro* result, the levels of GAP-43 mRNA increased in both PC12 cells with knockdown of KSRP and brains of KSRP KO mice. Using primary hippocampal cell cultures we found that partial knockdown or complete knockout of KSRP drastically increases axonal outgrowth, while overexpression stunts growth. The proper control of axonal outgrowth during development is critical for forming functional synapses, so it seems that careful control of both KSRP and HuD expression and their interaction with specific transcripts during differentiation are important for regulating axonal elongation and hence normal neuronal development.

Given that KSRP is highly expressed in brain tissue, it is interesting that there are relatively few studies examining how it affects neuronal development. One of these studies reported that the chicken KSRP homolog zip code-binding protein 2 (ZBP2) aids the other zip-code binding protein ZBP1 binding to β-actin mRNA in the nucleus, and shuttle the mRNA to neurites for localized translation during development [43]. ZBP1 expression has also been shown to be crucial for proper axonal localization of GAP-43 mRNA, as limited availability of ZBP1 depletes axonally localized GAP-43 and β-actin mRNAs and hampers axonal outgrowth [44]. Knock-down of mouse KSRP (mZBP2) using siRNAs blocked the initial outgrowth of neurites 12 hours after inducing differentiation of a mouse neuroblastoma cell line, presumably due to decreased loading of ZBP1 onto β-actin mRNA [43]. In the present study, we observed the opposite effect in primary cultures with depleted KSRP. This apparent discrepancy could be due to either differences in the neuronal cell type examined or the timing of the knockdown of KSRP expression, as we let the neurons grow for 3 DIV before performing shRNA-mediated knockdown. Thus, in this study KSRP was depleted from the hippocampal neurons after initiation of neurite growth whereas KSRP was depleted in the neuroblastoma cell line at early stages of differentiation [43]. Perhaps early in development KSRP may function to help load ZBP1 onto zip code containing mRNAs in the nucleus, while it can subsequently function in the cell body and processes to destabilize growth related transcripts to fine-tune neuronal development. It is important to note that the GAP-43 3′ UTR is also critical for its correct axonal localization [29] and effect on axonal elongation, as witnessed in our rescue study showing that overexpression of GAP-43 lacking these sequences (mCherry-GAP-γ-Actin3’) did not rescue the phenotype induced by KSRP overexpression. Therefore, the cytoplasmic function for KSRP that we demonstrate, particularly its selective rescue by axonally localizing GAP-43 mRNA, is a critical difference between our study and previous work on ZBP2 [43], which was focused on the nuclear function of this protein. Finally, the finding that knockdown of KSRP results in a similar mode of outgrowth as overexpression of axonally targeted GAP-43 mRNA [29], with decreased branching and increased elongation of axons, points to KSRP-mediated destabilization of axonal GAP-43 mRNA as an important determinant of this phenotype.

KSRP regulation of GAP-43 mRNA stability may not be the only mechanism affecting neuron differentiation and development. As shown by Winzen et al. [45] several transcripts bind KSRP in HeLa cell extracts including a number of mRNAs that encode for growth related proteins or signaling molecules related to cellular development. Furthermore, a recent study demonstrated that silencing KSRP in P19 cells induces the expression of some of very early markers of neuronal differentiation including nestin, IGF2, TUBB3, and ASCL1 [46]. Therefore, it will be important to identify the repertoire of KSRP target mRNAs and the role of this protein in the stabilization of these transcripts during neuronal differentiation. Although HuD and KSRP show similar expression patterns during brain development, i.e., they are highly expressed early in development and their levels drop off during adolescence [22], [39], there are differences in the specific cell types in which their expression persist in the adult brain. For instance, while the levels of HuD sharply decrease in hippocampal dentate granule cells (DGCs) after the first week of postnatal life [18], [39], KSRP protein is present in these cells until adulthood (data not shown). Along these lines it is important to note that in DGCs the temporal pattern of GAP-43 mRNA and protein expression closely matches that of HuD [39]. Interestingly, overexpression of HuD in adult DGCs rescues GAP-43 expression by increasing the stability of this mRNA [15], presumably by competing with KSRP, leading to an increased length of their axons [18]. These results suggest the ratio of HuD vs. KSRP in developing neurons may be critical for controlling GAP-43 mRNA levels, which in turn is a critical factor for determining axonal outgrowth [29].

In addition to their function in the CNS, both HuD and KSRP have been shown to localize to peripheral nerve axons via binding to SMN, and improper localization of these RBPs can affect motor neuron function [47]–[50]. Also, it is likely that the interplay of these proteins may be critical for the control of axonal regrowth after nerve injury. Examining how these two RNA-binding proteins, with opposing effects on mRNA stability, compete for ARE-containing mRNAs such as GAP-43 in axons will be the next step in understanding how they contribute to regulate axonal elongation.

## Supporting Information

Figure S1 **In vitro decay assays in the presence of a control RNA demonstrate consistent RNA recovery during the assays.** **A**. Capped and polyadenylated 32P-labeled GAP-43 mRNA was used for in vitro decay assays in the presence of GST or GST-KSRP as described in Bolognani et al., 2006 [15]. A non-polyadenylated and stable control RNA of smaller size (co RNA) was added the reactions to control for RNA yield after extractions. **B**. Decay curves show that RNA recovery does not change even when GAP-43 mRNA is destabilized in the presence of KSRP.
(TIF)

Figure S2 **Transfection of E17 neuronal cells with a different KSRP shRNA construct also results in increased axonal outgrowth.** **A**. Representative images of GFP-expressing cells transfected for 48 hours with either GFP-sh-control or GFP-sh2-KSRP plasmids. **B**. Results from quantitation of axonal length using Neurolucida. *p<0.05 (n = 8 separate slides counting 4–6 cell per slide).
(TIF)

Figure S3 **Western blot analysis of KSRP protein expression in E17 cortices from wild type (Ksrp^+/+^), heterozygous (Ksrp^+/−^) and KO (Ksrp^−/−^) embryos.** Total homogenates were prepared from E 17 cortices from wild type, heterozygous and KSRP KO mice and western blots run as described in Bolognani et al., 2006 [15] using increasing amounts of total protein. Blots were first probed with KSRP/FBP2 antibodies (1∶1000 dilution). and re-probed with GAPDH antibodies (1∶5000). Note that the levels of KSRP in heterozygous mice are about 15% of the levels in wild type embryos.
(TIF)
