# Supplementary figures and images for: KSRP Modulation of GAP-43 mRNA Stability Restricts Axonal Outgrowth in Embryonic Hippocampal Neurons
Source: PLoS One. 2013 Nov 14;8(11):e79255. doi: 10.1371/journal.pone.0079255 (PMC3828348; doi:10.1371/journal.pone.0079255)

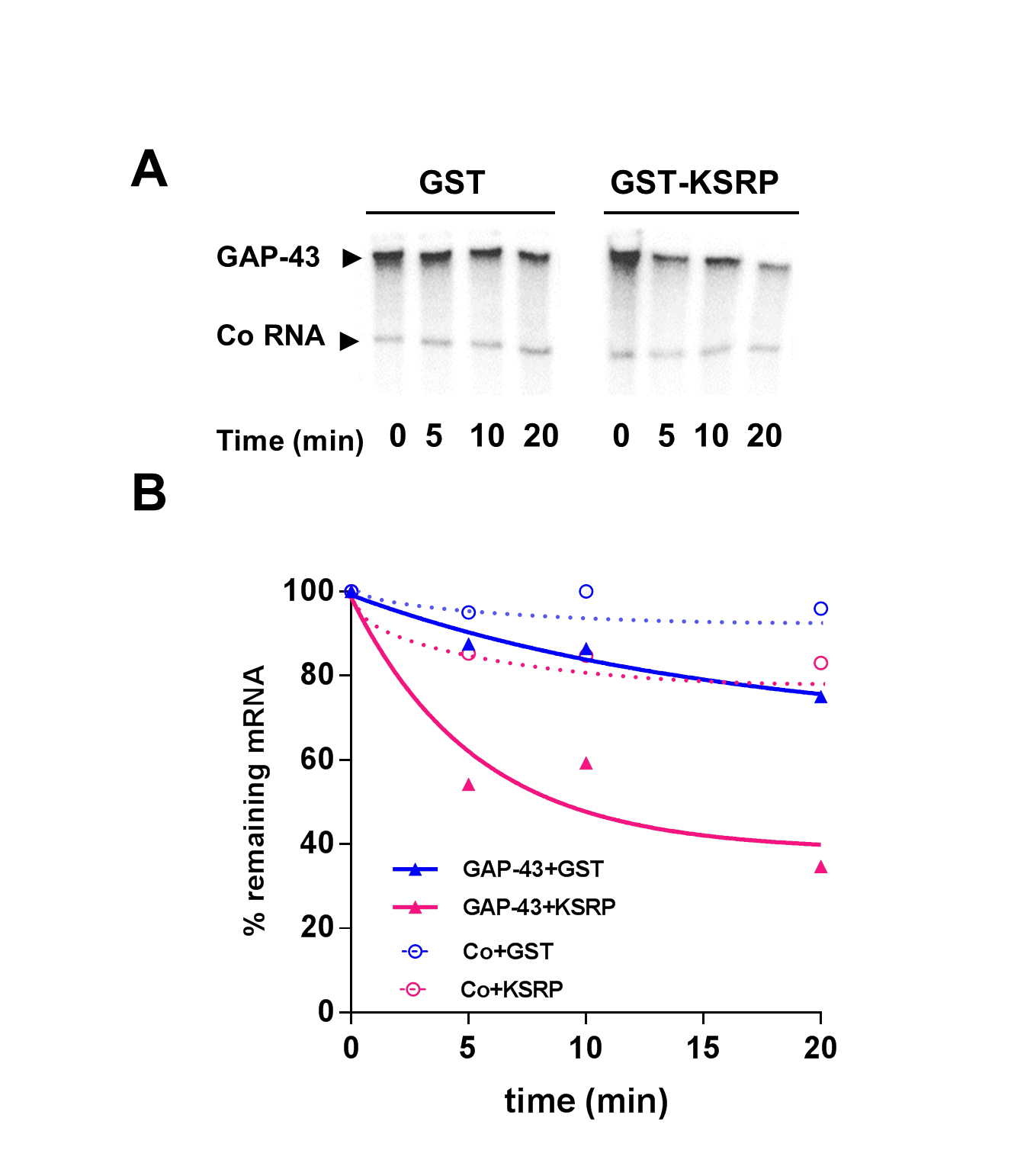

Supplement: Figure S1 — In vitro decay assays in the presence of a control RNA demonstrate consistent RNA recovery during the assays. A. Capped and polyadenylated 32P-labeled GAP-43 mRNA was used for in vitro decay assays in the presence of GST or GST-KSRP as described in Bolognani et al., 2006 [15]. A non-polyadenylated and stable control RNA of smaller size (co RNA) was added the reactions to control for RNA yield after extractions. B. Decay curves show that RNA recovery does not change even when GAP-43 mRNA is destabilized in the presence of KSRP. (TIF) [file pone.0079255.s001.tif]

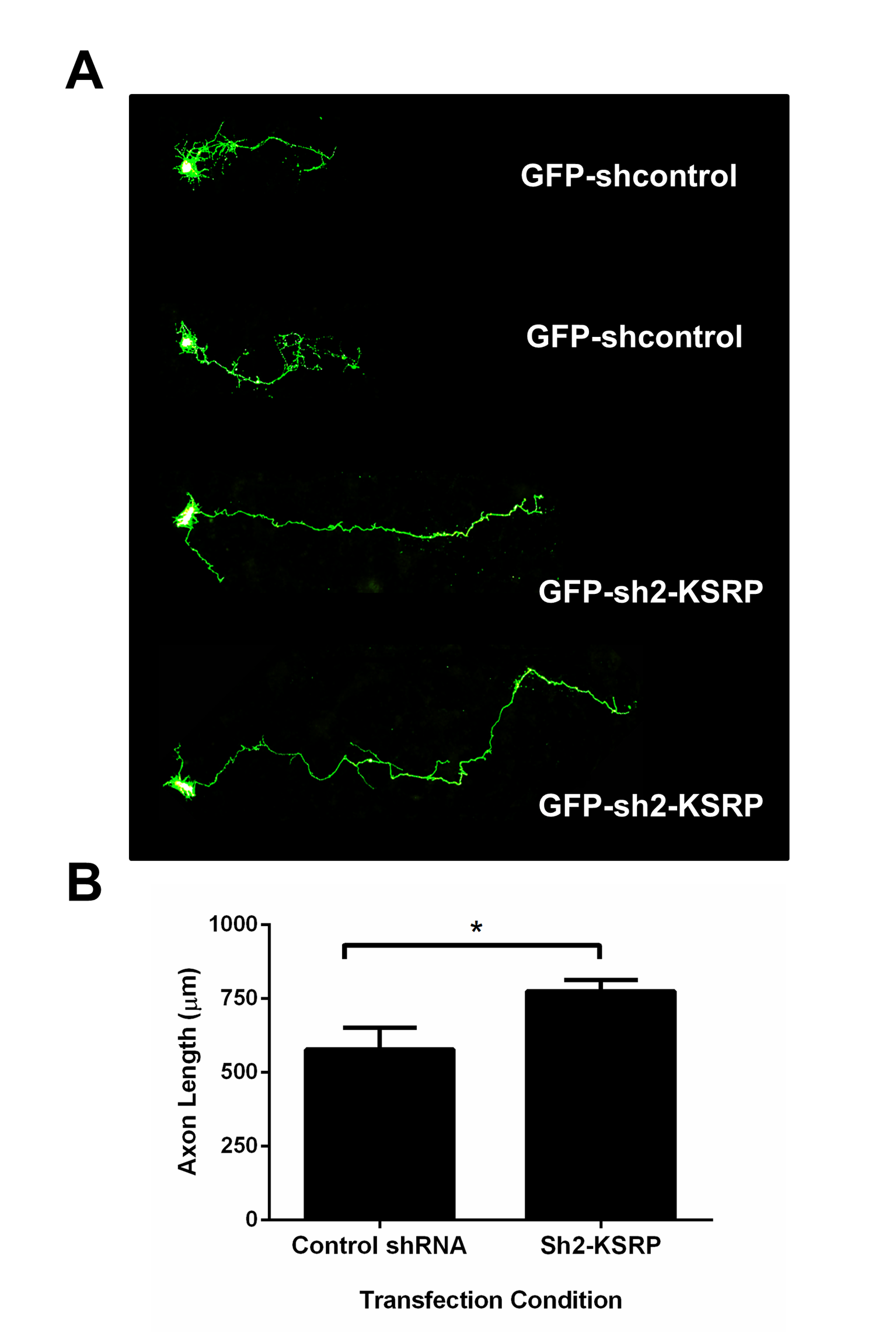

Supplement: Figure S2 — Transfection of E17 neuronal cells with a different KSRP shRNA construct also results in increased axonal outgrowth. A. Representative images of GFP-expressing cells transfected for 48 hours with either GFP-sh-control or GFP-sh2-KSRP plasmids. B. Results from quantitation of axonal length using Neurolucida. *p<0.05 (n = 8 separate slides counting 4–6 cell per slide). (TIF) [file pone.0079255.s002.tif]

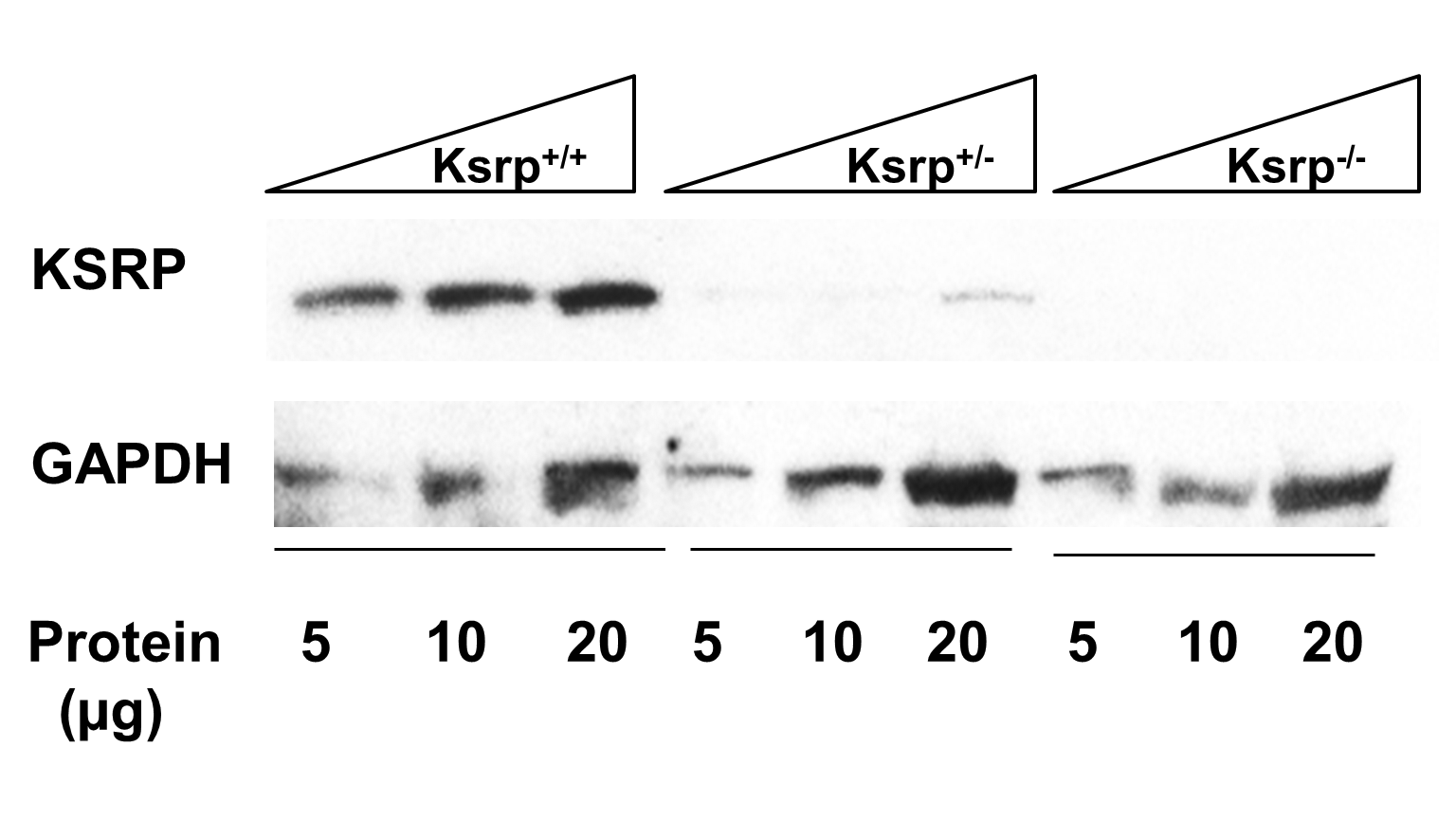

Supplement: Figure S3 — Western blot analysis of KSRP protein expression in E17 cortices from wild type (Ksrp+/+), heterozygous (Ksrp+/−) and KO (Ksrp−/−) embryos. Total homogenates were prepared from E 17 cortices from wild type, heterozygous and KSRP KO mice and western blots run as described in Bolognani et al., 2006 [15] using increasing amounts of total protein. Blots were first probed with KSRP/FBP2 antibodies (1∶1000 dilution). and re-probed with GAPDH antibodies (1∶5000). Note that the levels of KSRP in heterozygous mice are about 15% of the levels in wild type embryos. (TIF) [file pone.0079255.s003.tif]
